# Alterations of Bone Metabolism in Patients with Diabetes Mellitus

**DOI:** 10.1155/2019/5984681

**Published:** 2019-12-22

**Authors:** Sain S. Safarova

**Affiliations:** Department of Internal Medicine, Azerbaijan Medical University, Baku AZ1000, Azerbaijan

## Abstract

**Aim:**

The study aims to develop a practical model for screening bone turnover state in patients with diabetes and evaluate its clinical usefulness to identify diabetic osteopathy.

**Materials:**

The study was conducted in 2015–2017 in the Endocrinology Department of the Therapeutic Clinic of AM University. A total of 235 patients were assessed in the study (98 with T1DM and 137 with T2DM). 89 nondiabetic subjects served as controls. Bone mineral density (BMD) [by dual energy X-ray absorptiometry (DXA)] and serum markers of bone remodeling [aminoterminal propeptide of procollagen type I (P1NP) and c-terminal telopeptide of type I collagen (CTX)], parathyrin, and 25(OH)D were measured in all 235 patients.

**Results:**

Our results show that patients with T2DM have lower b-CTx values and relatively higher levels of P1NP, reflecting less pronounced changes in bone metabolism compared to patients with T1DM, regardless of age or duration of the disease. Osteoporosis was detected in 50% of patients with T1DM, compared to 13% of patients with T2DM.

**Conclusion:**

In some cases, bone remodeling markers are useful for improving the assessment of the state of bone tissue in early stages of diabetes, while alterations in bone microarchitecture may not always be captured by bone mineral density measurements.

## 1. Introduction

Diabetes-related complications are a consequence of severe metabolic disorders in the body. One of the most socially significant ones is diabetic osteopathy, which increases the risk of fractures, leading to a high level of disability and mortality [1]. Statistics suggest the incidence of femoral neck fractures in people with diabetes is six times higher than in the general population [1, 2]. As a result of insulin secretion deficiency, bone formation slows down in T1DM, while bone resorption becomes relatively faster, leading to a decrease in bone mass density, impaired mineralization, and bone microarchitecture [2]. Changes in bone tissue metabolism in patients with type 2 diabetes occur somewhat differently [3, 4]. The risk of developing fractures is 10–30% higher in T2DM patients than in those without diabetes that were matched for age [4–6]. Age-related bone loss increases the risk of fracture in the geriatric population (≥65 years). The risk of bone fractures remains high even after eliminating factors such as sensorimotor deficiency and neuropathy that contribute to a fall [4].The paradox of fragility fractures at T2DM is the high bone mineral density (BMD) in most of the currently published research, but, at the same time, there is a contrasting decrease in bone micro- and microarchitecture quality [3]. This complicates proper screening of this category of patients at a high risk of fractures.

The purpose of this study is to develop a practical model for screening bone turnover state in patients with type 1 and type 2 diabetes and evaluate the model's clinical usefulness in identifying diabetic osteopathy.

## 2. Materials and Methods

The research was conducted in accordance with the principles of the Helsinki Declaration and was approved by the Health Research Ethics Committee of AM University. After an explanation of the aim of the study, written informed consent was received from each participant.

A crosssectional study included 98 (female: 57/male: 41) patients with T1DM and 137 (female: 85/male: 52) patients with T2DM, who had not been diagnosed with osteoporosis previously. The age of surveyed patients was between 40 and 70 years (55.8 ± 0.7 for T1DM and 58.4 ± 0.9 for T2DM). Duration of diabetes: 16.6 ± 0.6 for T1DM and 8.1 ± 0.7 for T2DM, with the mean value of 57 ± 0.2 HbA1c for T1DM and 58 ± 0.4% HbA1c for T2DM; neuropathy and retinopathy were detected in 42% and 88% of patients with DM.

### 2.1. Exclusion Criteria

Patients who have been treated with steroid, glitazones, and type 2 sodium-glucose cotransporter (SGLT-2) inhibitors; treated for osteoporosis or have a history of fracture; and patients with acute complications of diabetes, hepatic dysfunction, renal dysfunction, and diabetic nephropathy of the 4-5 stages in the anamnesis were excluded. The control group comprised 82 patients (female: 48/male: 34; 55.9 ± 0.9 years). Apparently healthy, normoglycemic subjects were recruited as controls. Control group's BMI was 28.7 ± 0.4 kg/m^2^.

Height and weight were measured with standardized techniques. BMI was expressed as weight per height squared (kg/m^2^). Blood samples were drawn before 10 a.m.; they were put into heparin for subsequent centrifugation, stored at −70°C, and thawed immediately before serum biomarker and hormonal analyses. Biochemistry panel, including HbA1c, sodium, potassium, magnesium (Mg^2+^), total calcium (tCa), ionized calcium (Ca^2+^), phosphate (P^+^), creatinine, albumin, alkaline phosphatase (ALP), aminoterminal propeptide of procollagen type I (PINP), and C-terminal telopeptide of type I collagen (beta-CTx) in serum, was measured using an automatic electrochemiluminescence analyzer (COBAS C, Roche Diagnostics GmbH Mannheim, Germany). Glomerular filtration rate (GFR) was calculated by the CKD-EPI equation: (=141 × min (SCr (smg/dl)/k, 1)a × max (SCr/k,1)−1,209 × 0.993 age (x1.018 if female) (in ml/min/1.73 m^2^). Commercially available ELISA assays of insulin, parathyroid hormone (PTH), calcitonin (CT), and vitamin D (25(OH)D) were performed according to the manufacturer's instructions. Insulin sensitivity was determined by the homeostasis model assessment of insulin resistance (HOMA-IR) using the following equation: (fasting insulin (mIU/ml) × fasting glucose (mmol/L))/22.5.

All subjects underwent DXA on a densitometer (DXA HOLOGIC, Discovery QDR 4500A, USA) for the lumbar spine, proximal, and femoral neck areas. The World Health Organization criteria for diagnosis of osteoporosis are defined by BMD (T-score ≤ 2.5 SD), osteopenia (T-score from −1 to −2.5 SD), and normal (T-score > −1).

The statistical analysis was carried out using the STATISTICA 10 program. Data were presented as mean (M) and confidence interval (95% CI), unless specified otherwise. Statistical analysis was performed using unpaired parametric data analyzed by the Mann–Whitney *U* test. Spearman's rank correlation was calculated to assess the power of connection between the parameters. A value of *p* < 0.05 was considered statistically significant.

## 3. Results

### 3.1. Clinical Parameters in the Three Groups

All participants were Caucasians; the mean age was 55.8 ± 0.7 for T1DM patients, 58.4 ± 0.9 for T2DM patients, and 55.9 ± 0.9 for controls. Mean BMI was 26.1 ± 0.2 kg/m^2^ (T1DM), 30.0 ± 0.3 kg/m^2^ (T2DM), and 28.7 ± 0.4 kg/m^2^ (controls). The differences in clinical parameters between these groups are illustrated in Table 1.

Ca^2+^ was significantly lower in the DM groups than in the control group (*p* < 0.05 and *p* < 0.001). Overall, females had a lower incidence of Ca^2+^ than males. Except for magnesium (Mg^2+^) and potassium (K^+^), the remaining clinical parameters (including phosphorus) were not significantly different among the three groups. Serum Mg^2+^ levels were noticeably decreased in both groups with diabetes (*p* < 0.05 and *p* < 0.01). Hypomagnesemia was detected in 13% of patients with type 1 diabetes and 11% of patients with T2DM. Moreover, a change in the Mg^2+^ content was 1.5–2 times more frequent in women than in men. HOMA-IR was prominently higher in T2DM patients than in the control group (*p* < 0.005 and *p* < 0.05). GFR was significantly higher in the T2DM group than in the T1DM group (*p* < 0.05); while albumin was significantly lower in T1DM than in T2DM (*p* < 0.05).

### 3.2. Calcium-Regulated Hormones, Plasma Levels of Bone Turnover Markers, and DXA

The level of PTH among groups of patients with T1DM and T2DM was slightly higher than the values of this indicator in the control group (*p* < 0.05). In the T1DM group, men had lower PTH levels than women, while in T2DM men had higher PTH levels than women. There was a significant difference in 25(OH)D levels between all DM patients and controls. As a result of data analysis, higher serum values of calcitonin were found in patients with T1DM compared to those of T2DM patients and the control group. The DM group women also had 12% higher CT levels than men. Characteristics of calcium-regulating hormones, bone markers, and BMD values of patients with type 1 and type 2 diabetes mellitus and the control group are shown in Table 2.

The bone turnover markers PINP and b-CTX were higher in both DM groups than in controls. P1NP was also considerably lower in the T1DM group compared to T2DM. There was a difference in b-CTX levels between all DM patients and controls (*p* < 0.05), but not at the ALP level. ALP level in the groups of patients with diabetes was comparable with the control group (*p* > 0.05). The level of P1NP is statistically reduced in patients with type 1 diabetes in comparison with the control group (*p* < 0.05). The comparison of BMD values between patients with type 1 and type 2 diabetes mellitus and the control group is shown in Table 2.

Results of the analysis of the T-score have found that group bone density for the L1–L4 and FN areas was reduced in T1DM in comparison with the control group (*p* < 0.001), with no such differences between T2DM and control groups (*p* < 0.005). Bone density in these areas was significantly lower in females than in males with type 1 diabetes, while T2DM showed no such statistical difference between genders (*p* < 0.005 and *p* < 0.05). However, when patients with DM were compared with those of the same sex in the control group, the lower T-score values at the L1–L4 area were found in males with T1DM (*p* < 0.001) and T2DM (*p* < 0.005). At the same time, no substantial differences were determined in T-score BMD for the L1–L4 area between women with T2DM and the control group (*p* > 0.01); T-score for the FN area in women with T2DM was lower in comparison with the control group (*p* < 0.05).

## 4. Discussion

In diabetes, there are multiple factors that increase the risk of bone turnover disorders. Therefore, we compared biochemical markers of bone metabolism in the same-aged patients, both healthy and with diabetes. The institutionalized group comprised only patients with well-controlled diabetes without late-stage complications.

There was some difference in the serum concentration of vitamin D between the three groups of patients, where vitamin D acted by stimulating intestinal absorption of calcium and phosphorus [5], as evident by the correlation between the Ca^2+^ and 25(OH)D level for T1DM (*R* = 0.507; *p*=0.001); for T2DM (*R* = 0.277; *p*=0.01).Consequently, vitamin D regulates calcium and phosphorus homeostasis.

Low levels of Mg^2+^ can reduce the activity of PTH, reducing the synthesis of alpha 1-hydroxylase. This in turn reduces the concentration of the active form of vitamin D and Ca^2+^in serum, adversely affecting the metabolism of the mineral component of the bone and changing the structure of hydroxyapatite crystals and the overall architecture of the bone [2]. A positive correlation was found between Mg^2+^ and vitamin D for T1DM (*R* = 0.516; *p*=0.002) and for T2DM (*R* = 0.302; *p*=0.03), as well as the positive relationship between Mg^2+^ and Ca^2+^ for T2DM (*R* = 0.321; *p*=0.01).

In patients with diabetes, a statistically positive relationship was found between albumin and GFR for T1DM (*R* = 0.264; *p*=0.04) and for T2DM (*R* = 0.283; *p*=0.01). Additionally, a significant negative correlation was determined between albumin and b-CTx levels for T1DM (*R* = −0.330; *p*=0.01) and for T2DM (*R* = −0.387; *p*=0.001). Hypoalbuminemia can directly and indirectly influence the bone metabolism and diminish transfer of minerals in bone tissue, resulting in reduced formation of hydroxyapatite crystals, which also affects the metabolism of PTH and vitamin D [6].

A major negative association was determined between GFR and b-CTx for T1DM patients with longer duration of diabetes (*R* = −0.204; *p*=0.04) and for T2DM (*R* = −0.203, *p*=0.01).

The association between bone remodeling markers and renal function is correlated with the ability of the kidneys to eliminate them, thereby clearing the bloodstream and, therefore, decreased GFR [6]. For example, a decreased GFR will reduce the urinary excretion of CTX, and, therefore, increase serum levels. Research has shown that GFR significantly correlates with PTH in T2DM (*R* = −0.213; *p*=0.04), as well as GFR and vitamin D (*R* = 0.346; *p*=0.001). As a consequence of renal function decrease, phosphate retention contributes to the secondary hyperparathyroidism development via a combination of interlinked factors [4].

The absolute lack of insulin secretion leads to PTH hypersecretion, associated with a decrease in serum Ca^2+^ levels and causes a secondary hyperparathyroidism. This contributes to decalcification of bones, which is consistent with data from several other studies [7]. Furthermore, the serum PTH level in T2DM patients was associated with the HOMA-IR index (*R* = −0. 273, *p*=0.01), which confirms the effect of PTH on insulin secretion from b-cells and its effect on glucose metabolism [8].

A correlation association was found between increases in serum calcitonin and duration of diabetes (*R* = 0.638; *p*=0.001) for T1DM and for T2DM (*R* = 0.430, *p*=0.001).A positive linear relationship was established between the level of CT and the HOMA-IR index with increased duration of T2DM (*R* = 0.615; *p*=0.03); thus, confirming previous studies, which detected that calcitonin inhibits glucose-stimulated insulin release. Evidently, CT leads to decreased insulin sensitivity of muscles and adipose tissue and increases glycogenolysis and peripheral insulin resistance [7].

Lower P1NP level in patients with type 1 diabetes in comparison with the control group indicated a major reduction in bone formation. ALP did not show any significant difference between the DMs and the controls. One possible explanation for the differences between bone formation markers (ALP/PINP) in diabetes may be that they reflect different aspects of osteoblast function and bone formation. While ALP is produced by mature osteoblasts, P1NP is formed during the procollagen synthesis phase [9–11].

The mean values of the bone resorption marker b-CTx in both DM groups were in reference intervals, but higher than in the control group, which indicates increased bone resorption in accordance with the previously reported literature [1]. Also, the results of the analysis revealed a higher BMI in individuals with low serum b-CTx, as described previously in several other studies [4]. A statistically negative relationship was found between HbA1c and P1NP for T1DM (*R* = −0.252; *p*=0.03) and for T2DM (*R* = −0.254; *p*=0.01). The negative association indicated that increases of blood glucose concentration may affect bones by altering bone formation process; hence, individuals with diabetes are at higher risk of fragility fractures [7, 10–12].

Analysis of bone density has identified that patients in both DMs had an increased risk of bone fractures for the lumbar spine T-score (64% for T1DM and 44% for T2DM; 26% for the controls) and femoral neck area (41% for T1DM and 36% for T2DM; 22% for the controls). Furthermore, there was a lower risk of bone fracture for the proximal femur area (36% for T1DM and 31% for T2DM; 20% for the controls). These findings are consistent with the results obtained in other studies [1, 4].The reduced bone mineral content, which depended on the duration of diabetes, was pronounced most in patients under the age of 50, especially in men.

Our study has several strengths. Firstly, this study reports an inverse association between serum electrolytes, hormones levels, and BMD in patients with T1DM and T2DM. The crosssectional design used in this study measured the investigated parameters in a single time point only, imposing limitations to the extent of the study. The significance of our findings may be limited by the small sample size; some of the results reported may be flawed by insufficient data access. Hence, some key statistics could not be measured further, which may affect the selection of controls. We collected the serum samples from all the participants once, and BMD was detected once at each anatomical site; this may have caused deviations in some variables (bone remodeling marker levels and BMD values).

## 5. Conclusion

The results of this study show that patients with T2DM had lower b-CTx values and relatively higher levels of P1NP, reflecting less pronounced changes in bone metabolism compared with T1DM patients, regardless of age and duration of the disease. Research suggests that in patients with T1DM (50%), osteoporosis was detected more frequently than in T2DM (13%). Biochemical markers and bone density can detect disorders in skeletal metabolism. However, bone remodeling markers can be useful in some cases to improve the assessment of the state of bone tissue, when the BMD measurement does not reflect the actual tendency, for example, in the early stages of T2DM. In some cases, bone remodeling markers are useful for improving the assessment of the state of bone tissue in early stages of diabetes, while alterations in bone microarchitecture may not always be captured by bone mineral density measurements.

## Figures and Tables

**Table 1:** Clinical parameters in the groups.

Variables	DM1 (*n* = 98)	DM2 (*n* = 137)	Control (*n* = 82)
Age, years	55.8 (54.4–57.3)	58.4 (57.3–59.5)	55.9 (54.2–57.7)
Sex, male : female	41 : 57	52 : 85	39 : 43
BMI, kg/m^2^	26.1 (25.6–26.5)	30.0 (29.4–30.6)	28.7 (27.9–29.5)
Duration of DM, years	16.6 (15.4–17.8)	8.1 (7.2–8.8)	
HbA1c, mmol/mol	57 (54–62)	58 (55–62)	30 (28–31)
Calcium^2+^, mmol/L	1.09 (1.07–1.11)^*∗*^	1.06 (1.03–1.08)	1.13 (1.11–1.15)
Phosphate, mmol/L	5.4 (5.2–5.6)^*∗*^	4.9 (4.7–5.1)	5.1 (4.9–5.2)
Magnesium, mmol/L	0.63 (0.57–0.68)^*∗*^	0.63 (0.6–0.67)	0.72 (0.66–0.78)
Potassium, mmol/L	4.4 (4.2–4.6)^*∗*^	4.3 (4.1–4.4)	4.3 (4.1–4.5)
Sodium, mmol/L	142.2 (140.6–143.8)^*∗*^	140.9 (139.6–142.3)	138.5 (137.2–139.6)
Creatinine, umol/L	72.5 (69.85–76.04)^*∗*^	69.85 (67.2–72.5)	66.32 (63.66–68.97)
eGFR, mL/min/1.73	87.9 (84.1–91.7)^*∗*^	88.5 (85.4–91.5)^*∗*^	95.2 (91.8–98.6)
Albumin, g/L	42 (41–43)^*∗*^	43 (41–44)^*∗*^	45 (43–46)

^*∗*^ *p* < 0.05 compared with the control group data.

**Table 2:** Characteristics of calcium-regulating hormones, bone markers, and BMD values.

Variables	DM1 (*n* = 98)	DM2 (*n* = 137)	Control (*n* = 82)
PTH, ng/L	51.16 (47.17–55.13)^*∗*^	51.69 (48.82–54.56)^*∗*^	45.09 (40.38–49.79)
CT, ng/L	12.07 (9.75–14.38)^*∗*^	10.23 (8.48–11.62)^*∗*^	5.5 (4.19–6.84)
25 (OH) D, nmol/L	60.12 (53.21–67.04)^*∗*^	62.69 (57.35–68.08)^*∗*^	75.9 (67.26–84.51)
ALP, IU/L	118.3 (110.1–126.4)	122.2 (116.2–128.1)	123.5 (113.8–133.2)
P1NP, ng/mL	40.58 (37.18–43.98)^*∗*^	42.08 (39.81–44.35)	47.09 (42.82–51.35)
b-CTX, ng/mL	0.525 (0.468–0.582)^*∗*^	0.495 (0.456–0.533)^*∗*^	0.424 (0.383–0.466)
Т-score (L1–L4)	−2.04 (−2.3; −1.7)^*∗*^	−1.08 (−1.3; −0.8)^*∗*^	−0.73 (−1.1; −0.3)
Т-score (prox.)	−1.44 (−1.7; −1.1)^*∗*^	−0.95 (−1.2; −0.7)^*∗*^	−0.49 (−0.8; −0.1)
Т-score (FN)	−1.68 (−1.9; −1.3)^*∗*^	−1.12 (−1.3; −0.8)^*∗*^	−0.64 (−1.0; −0.2)

^*∗*^ *p* < 0.05 compared with the control group data.

## Data Availability

The clinical material data used to support the findings of this study are available from the corresponding author upon request.
